# Triage Algorithms for Mass-Casualty Bioterrorism: A Systematic Review

**DOI:** 10.3390/ijerph20065070

**Published:** 2023-03-13

**Authors:** Feida Zhao, Chao Zhao, Song Bai, Lulu Yao, Yongzhong Zhang

**Affiliations:** 1 Institute of Disaster and Emergency Medicine, Tianjin University, Tianjin 300072, China; 2 Center for Biosafety Research and Strategy, Tianjin University, Tianjin 300072, China; 3 Evaluation and Optimization of Health Emergency Response Capacity, Institute of Disaster and Emergency Medicine, Tianjin University, Tianjin 300072, China; 4 Emergency Medicine, Institute of Disaster and Emergency Medicine, Tianjin University, Tianjin 300072, China; 5 Epidemiology and Health Statistics, Institute of Disaster and Emergency Medicine, Tianjin University, Tianjin 300072, China

**Keywords:** triage, triage algorithm, bioterrorism, terrorism, anthrax, psychosocial

## Abstract

Objectives: To understand existing triage algorithms, propose improvement measures through comparison to better deal with mass-casualty incidents caused by bioterrorism. Study Design: Systematic review. Methods: Medline, Scopus and Web of Science were searched up to January 2022. The studies investigating triage algorithms for mass-casualty bioterrorism. Quality assessment was performed using the International Narrative Systematic Assessment tool. Data extractions were performed by four reviewers. Results: Of the 475 titles identified in the search, 10 studies were included. There were four studies on triage algorithms for most bioterrorism events, four studies on triage algorithms for anthrax and two studies on triage algorithms for mental or psychosocial problems caused by bioterrorism events. We introduced and compared 10 triage algorithms used for different bioterrorism situations. Conclusion: For triage algorithms for most bioterrorism events, it is necessary to determine the time and place of the attack as soon as possible, control the number of exposed and potentially exposed people, prevent infection and determine the type of biological agents used. Research on the effects of decontamination on bioterrorism attacks needs to continue. For anthrax triage, future research should improve the distinction between inhalational anthrax symptoms and common disease symptoms and improve the efficiency of triage measures. More attention should be paid to triage algorithms for mental or psychosocial problems caused by bioterrorism events.

## 1. Background

Mass-casualty incidents (MCIs) are defined as incidents that lead to a surge of patients that overwhelms the local health-care system [1,2]. Mass casualty incidents are increasing in frequency throughout the United States [3]. The most common reason for MCIs is natural disasters [4]. Other reasons include accidents or certain intentional events [3]. Therefore, the current research on triage tools focuses on these circumstances. However, the frequency of terrorist incidents has increased in recent years, creating new challenges [5].

Terrorism, in its broadest sense, is the use of violence and fear to achieve an ideological aim. The term is used in this regard primarily to refer to intentional violence during peacetime or in the context of war against noncombatants (mostly civilians and neutral military personnel) [6].

Bioterrorism is terrorism that involves the intentional release or dissemination of biological agents. These agents include bacteria, viruses, insects, fungi or toxins and may be in a naturally occurring form or a human-modified form in much the same way as is seen in biological warfare [7]. Bioterrorism can be understood as making an attack using biological agents to achieve an ideological purpose. Bioterrorism may be favored because biological agents are relatively easy and inexpensive to obtain, can be easily disseminated and can cause widespread fear and panic beyond the actual physical damage.

Bioterrorism is not new and has been used for centuries [8]. Bioterrorism was already being used in the 14th century BCE, when the Hittites sent infected rams to their enemies [9,10]. Evidence has been produced that many countries developed secret biological weapons programs during World War I and World War II [11,12]. Recently, there are several biological attacks, such as the Rajneesh Cult that contaminated salad bars in Dalles, Oregon, with Salmonella typhimurium in 1984 [8] and a government employee of the US Army Research Institute for Infectious Disease who intentionally distributed anthrax spores through the US Postal Service in September 2001 [13,14].

Triage is defined as a set of procedures used to determine treatment and treatment priority according to a patient’s injury [15]. The purpose is to determine the treatment sequence of the wounded when there are not enough medical resources to treat all of the wounded and to reallocate resources so that more wounded individuals can be treated more efficiently [15,16,17].

Traditional triage algorithms have been mostly applied to trauma situations, and traditional triage algorithms determine the severity of patients by evaluating their mobility, respiratory rate, capillary filling, radial pulse and awareness. These categories are not relevant in bioterrorism attacks, unless the bioagent is combined with an explosive device [18]. However, after biological attacks, the typical symptoms of patients are fever or psychological problems. Some biological agents are extremely difficult to detect and do not cause illness for several hours to several days. Some bioterrorism agents, such as the smallpox virus, can be spread from person to person [19].

In summary, triage algorithms for bioterrorism are quite different from triage algorithms for trauma. Thus, there is a need to provide useful evidence and experience-based information for those who may become involved in mass-casualty bioterrorism. This systematic review aims to identify and describe the literature on triage algorithms that can be used in mass-casualty bioterrorism.

## 2. Methods

### 2.1. Search Strategy

Our systematic review involved the structured searching of Medline (via https://pubmed.ncbi.nlm.nih.gov/ (accessed on 25 January 2022)), Scopus (via https://www.scopus.com (accessed on 25 January 2022)) and Web of Science (via www.webofknowledge.com (accessed on 25 January 2022)). This review was performed according to the PRISMA guidelines (Supplementary Materials) [20]. The systematic search strategy is shown in Table 1.

### 2.2. Inclusion Criteria

- Specific triage algorithms for bioterrorism events caused by various biological agents.
- Triage algorithms that can be used in bioterrorism attacks regardless of the types of biological agents.
- Specific triage algorithms for mental or psychosocial problems caused by bioterrorism events.

### 2.3. Exclusion Criteria

- Articles without specific triage algorithms.
- Triage algorithms that do not apply only to bioterrorism.
- Articles that are not written in English.
- Articles without full texts.
- Letters to editors or conference papers without full texts.

### 2.4. Data Extraction

The retrieved titles were imported into Endnote software (EndNote X9.3.3). Duplicated titles were deleted, and the remaining titles were screened by the first authors. The abstracts of the remaining titles were evaluated by two reviewers. If these two individuals agreed that a title should be included, then the full-text article was assessed for eligibility. The full-text article assessments for eligibility were performed by another two reviewers. Discrepancies were resolved by discussion to reach a consensus.

### 2.5. Quality Assessment

A reviewer assessed the quality of the selected articles using the International Narrative Systematic Assessment (INSA) tool [21]. The INSA tool is used for assessing the quality of narrative reviews. Seven criteria are assessed; for each criterion, one point can be assigned. A review that is awarded a total of five points is considered good. Most articles are narrative in format; therefore, we used the INSA tool to assess the quality of the articles.

## 3. Results

**Figure 1:**
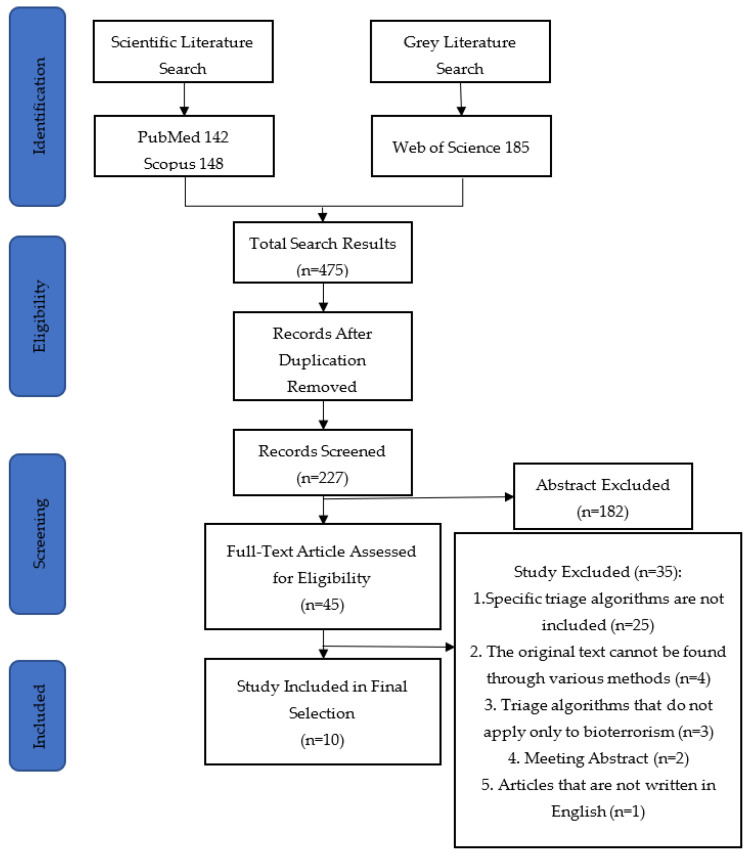
Flow diagram of the study selection process.

A total of 475 papers were identified in the electronic search, of which 10 were included in the final selection (Figure 1). There were four studies on triage algorithms for most bioterrorism events, four studies on triage algorithms for anthrax and two studies on triage algorithms for mental or psychosocial problems caused by bioterrorism events.

The total scores, basic information and data extracted from these ten articles were included in the final selection are shown in Table 2. There were five high-quality articles and five low-quality articles.

### 3.1. Triage Algorithms for Most Bioterrorism Events

The triage algorithms discussed in this section apply to most bioterrorism events, which means that all these triage algorithms can be used whether the biological agent is covert or not. These triage algorithms provide solid advice on how to deal with biological attacks. However, if there are triage algorithms that apply to specific biological agents, those specific triage algorithms should be used.

**Figure 2:**
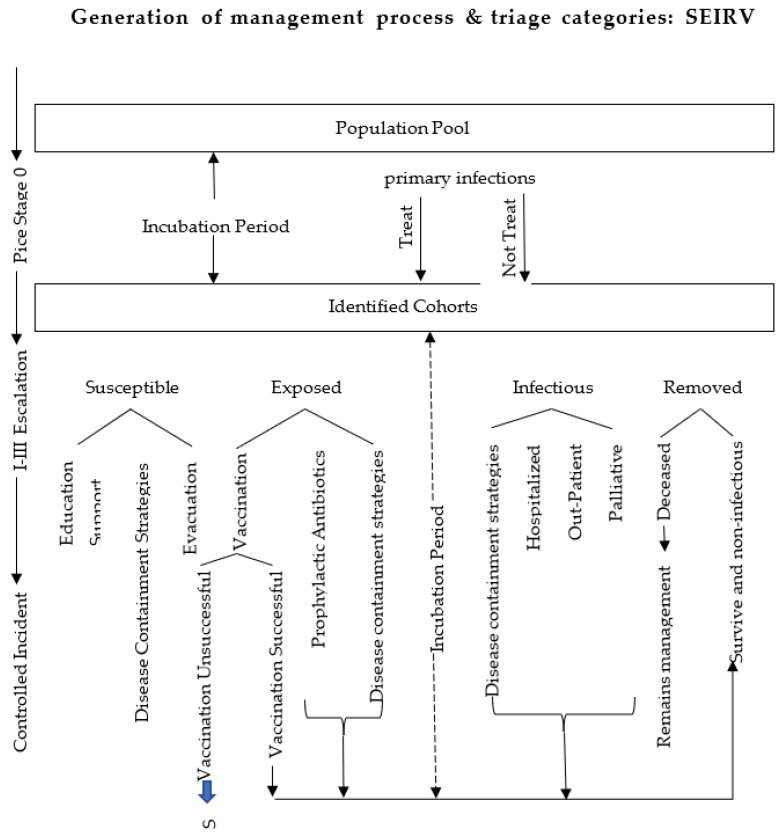
SEIRV model.

Burkle, F.M. proposed the SEIRV model according to epidemiological methods, which divides people into the following five categories. S: susceptible individuals (includes those with incomplete or unsuccessful vaccination); E: exposed individuals (those who are infected, incubating and noncontagious); I: infectious individuals (those who are symptomatic and contagious); R: removed individuals (those who are no longer sources of infection because they either survived or died from the illness, with the remains no longer contagious); and V: successfully vaccinated individuals (those with a confirmed ‘‘take’’ of a vaccine or who have completed the course for immunity) [22]. Figure 2 shows the SEIRV model.

**Figure 3:**
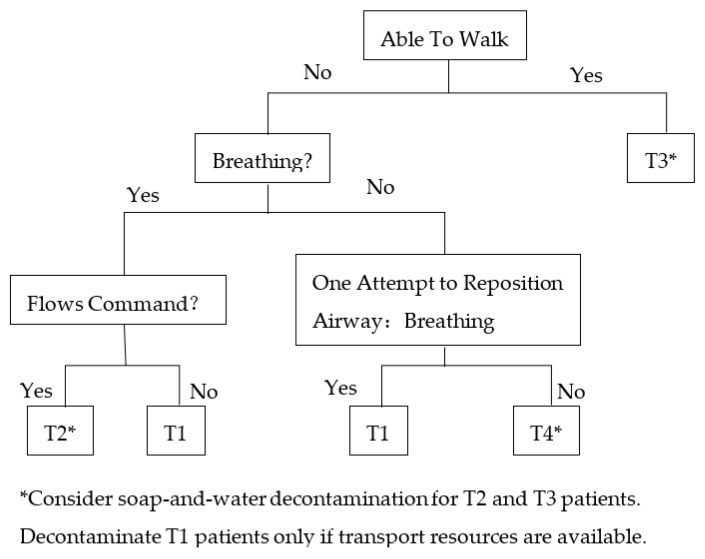
Triage algorithm based on START and the GCS.

Cone, D.C. proposed a triage algorithm for bioterrorism events according to the concepts of START (simple triage and rapid treatment) [30] and the GCS (Glasgow Coma Scale) [31], which are the most commonly used triage algorithms in the world. The proposed triage algorithm is different from the SEIRV model; it is suitable for use when the source of the biological agent is clear or if bioterrorism agents have been obviously released. The triage algorithm is shown in Figure 3 [18]. T1 means patients need immediate treatment, which is the first priority. Patients marked T2 are the second priority. Patients marked T3 are minor injuries, which is the third priority. Patients marked T4 are considered dead or cannot be treated with existing medical conditions, which is the fourth priority.

**Figure 4:**
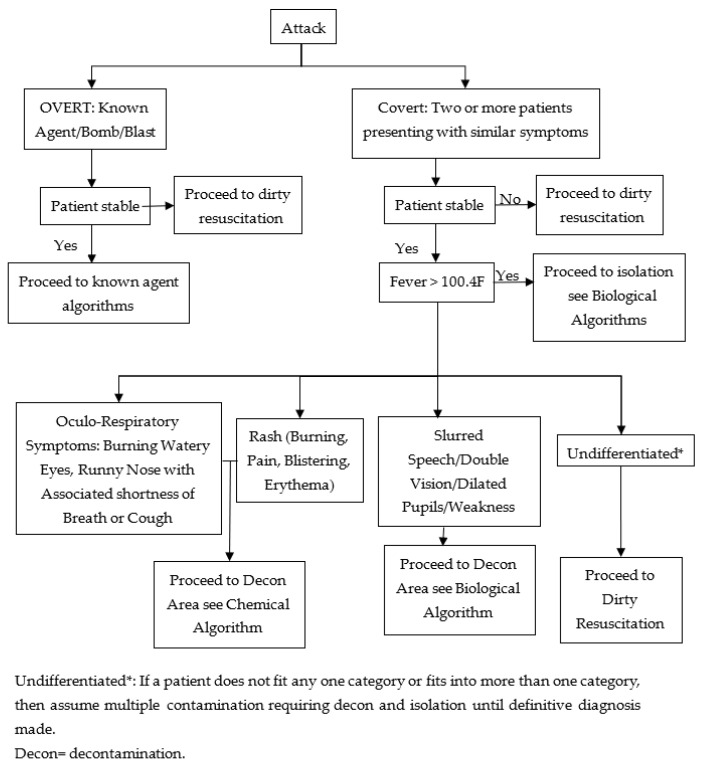
The attack algorithm.

Bond, W.F. designed a set of symptom-based, all-hazards, decision-making algorithms for a terrorist incident. There is an attack algorithm (Figure 4) that serves as the trunk algorithm for this algorithm. Terrorist attacks are divided into four different types according to the attack algorithm, and then four types of triage algorithms are designed for use [27]. Chemical, biological, radiological and nuclear (CBRN) contamination can render a healthcare treatment site inoperable; thus, decontamination initiation outside the patient treatment area is essential [32]. A decontamination area is an area where patients are decontaminated. Dirty resuscitation is a resuscitation of suspected contaminated, unstable patients limited to airway control, hemorrhage control, needle decompression, administration of antidotes and decontamination with soap and water [33].

**Figure 5:**
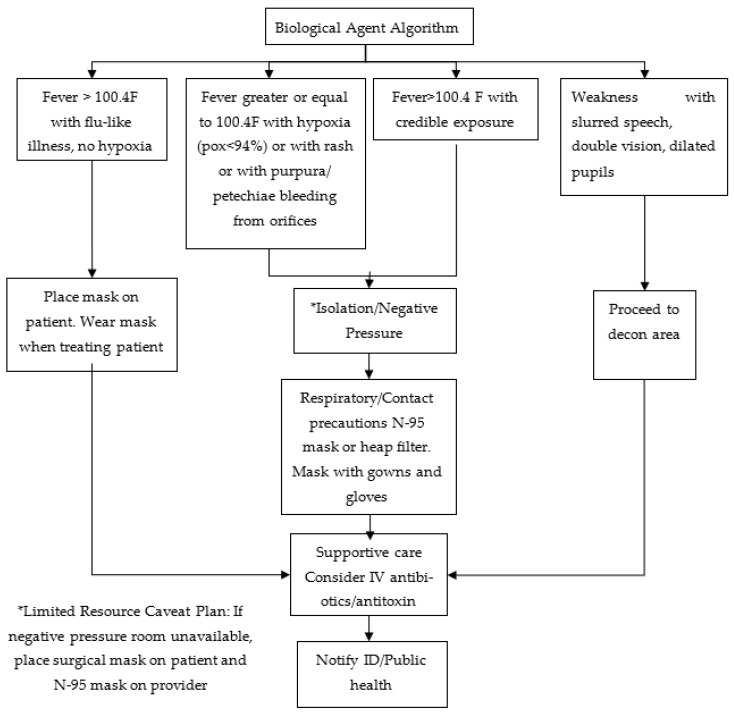
Triage algorithm under a bioterrorism attack.

Once an attack is confirmed as a bioterrorism attack, the subsequent triage algorithm related to a bioterrorism attack (Figure 5) is performed.

**Figure 6:**
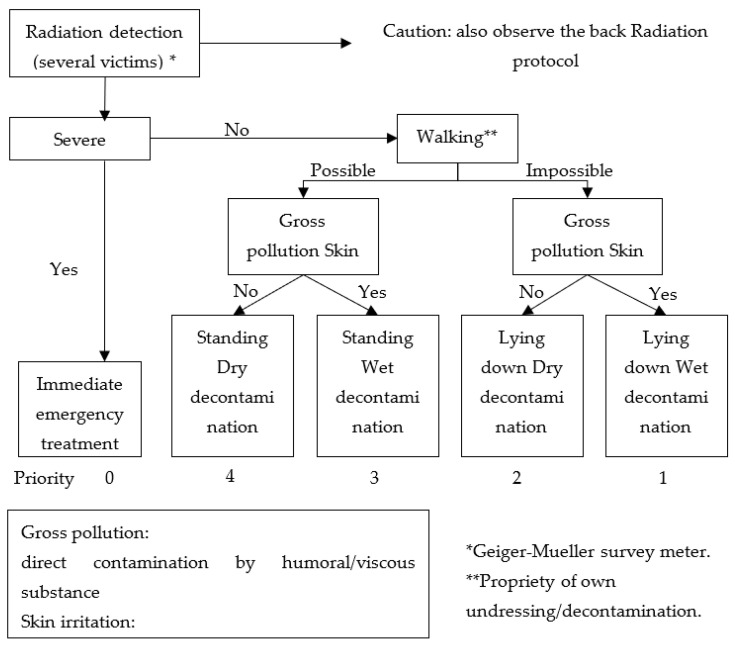
Flowchart for predecontamination triage.

The triage algorithm that is taught in the course of mass-casualty life support (MCLS) in Japan is shown in Figure 6. This algorithm was designed for responding to all types of chemical, biological, radiological/nuclear or explosive (CBRNE) disasters; however, the response based on the type of disaster is not emphasized [28]. The majority of cases use dry decontamination with undressing and the wiping of exposed sites only. Removal of the victim’s clothing may be the most important step in decontamination, as this can reduce the quantity of contaminant associated with the victim by an estimated 75% to 90% [34,35]. Wet decontamination is the cleaning of the skin. Solids can be physically removed by using a soft brush or towel, and the skin should be thoroughly washed with copious warm water and soap [36].

Among these four triage algorithms, the predecontamination triage algorithm proposed by Cone, D.C. in the Japanese mass-casualty life-support training course is the same as that proposed for ordinary mass-casualty incidents [37]. The wounded are divided into four different treatment priorities; however, the treatment measures for the four levels of wounded are not clear. Burkle, F.M.’s triage algorithm divides the wounded into four categories and takes different measures within the four categories of wounded. Bond, W. F.’s triage algorithm is based on the attack algorithm. After determining that no bioterrorism has occurred, this algorithm proposes special requirements for isolation measures to be taken during treatment and the equipment of medical staff and a triage algorithm to be used for boosterism.

### 3.2. Triage Algorithm for Anthrax

Anthrax is a serious infectious disease caused by Gram-positive, rod-shaped bacteria known as *Bacillus anthracis* [37]. Anthrax has the potential to pose a severe threat to public health; thus, it is categorized as a Category A biological agent by the U.S. Department of Health and Human Services and the U.S. Department of Agriculture. As it is easily extracted from soil around the world, anthrax is cheap and readily grown in large quantities and has long been part of the known bioweapon arsenals of several countries [38]. Anthrax can present as one of three types of infection in humans: inhalational, cutaneous and gastrointestinal [39].

Recently, another type of anthrax infection called injection anthrax has been identified in heroin-injecting drug users in northern Europe [38]. Given the rapid transit of food through the gastrointestinal system, it is unlikely that exposure to spores could cause gastrointestinal anthrax.

Instead, gastrointestinal anthrax likely results from the ingestion of poorly cooked meat that is contaminated with the germinated bacillus form of anthrax [26]. Inhaled anthrax is the most likely format to be used in a terrorist attack; this format is also the one with the highest mortality among the possible methods of anthrax attacks. Following a major attack, cutaneous anthrax cases might appear in conjunction with inhalational cases [40].

### 3.3. Cutaneous Anthrax

**Figure 7:**
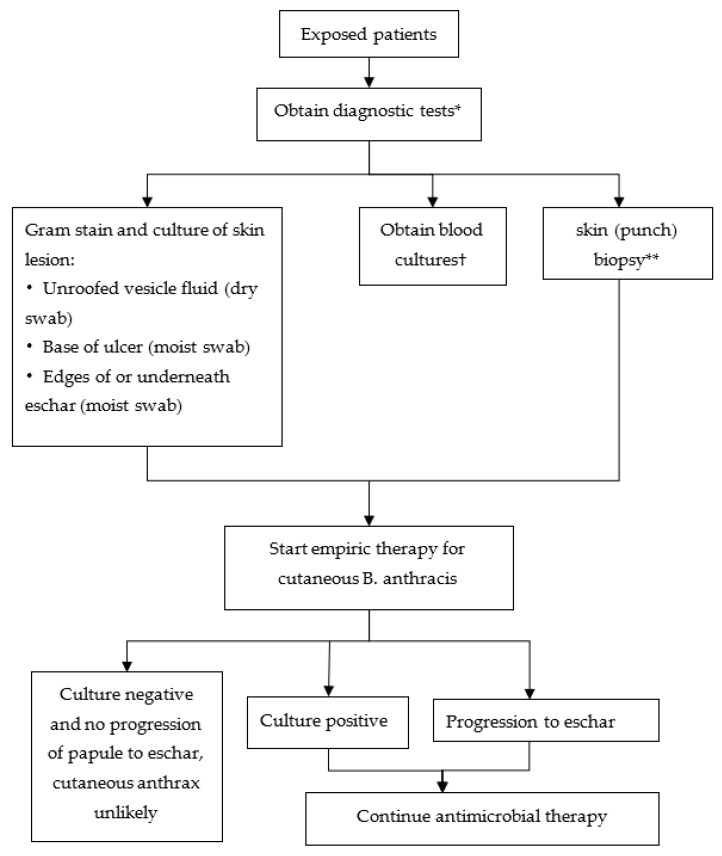
Clinical evaluation of persons with possible cutaneous anthrax. * Serologic testing available at the CDC may be an additional diagnostic technique for confirmation of cases of cutaneous anthrax. † If blood cultures are positive for *B. anthracis*, treat with antimicrobials as for inhalational anthrax. ** If patient is on antimicrobial drugs or if Gram stain and culture are negative for *B. anthracis* and clinical suspicion remains high. Punch biopsy should be submitted in formalin to CDC. Polymerase chain reaction can also be conducted on formalin-fixed specimens. Gram stain and culture are frequently negative for *B. anthracis* after initiation of antimicrobials.

Topical exposure to anthrax spores can result in cutaneous anthrax, especially in areas with previous cuts or abrasions [26]. Cutaneous anthrax is easier to detect, and the typical feature is the black eschar that is seen on affected areas [41]. The initial stage of cutaneous anthrax is similar to the painless papules produced by mosquito bites. The painless papules are generally produced 1–12 days after contact with spores [42]. Later, there is blister bleeding, turbidity and depression, accompanied by necrosis and ulcer formation. Black eschar presents 1 to 5 cm above the ulcer, surrounded by several cm of edema and erythema. As the characteristics of cutaneous anthrax are obvious, it is easy to judge cutaneous anthrax. The triage algorithm for cutaneous anthrax is shown in Figure 7.

Once the symptoms of cutaneous anthrax appear, it is possible to determine whether the wounded individual has been attacked by cutaneous anthrax by conducting Gram staining, the culture of skin lesions and blood cultures. Once either the skin progresses to eschar or the blood cultures are positive, then the individual is treated with antimicrobials; however, if the cultures are negative and there no progression of papules to eschar, then cutaneous anthrax is unlikely. Antimicrobial prophylaxis is continued for inhalational anthrax for 60 days if aerosol exposure to *B. anthracis* is known or suspected.

### 3.4. Inhalational Anthrax

In terms of its use as an organism of bioterrorism, anthrax is most likely to be delivered in its spore form as an inhaled agent [43]. When a person breathes in anthrax spores, they can develop inhalational anthrax [38]. Infection usually develops within a week after exposure; however, this development can take up to 2 months.

Without treatment, only approximately 10–15% of patients infected with inhalational anthrax survive. However, with aggressive treatment, approximately 55% of patients survive [38]. Early diagnosis is difficult and requires a high degree of suspicion [39]. The clinical presentation of inhalational anthrax occurs in two stages [26]. At first, physical findings are usually nonspecific [40] and include fever, dyspnea, cough, headache, vomiting, chills, weakness, abdominal pain and chest pain. The second stage develops abruptly and includes sudden fever, dyspnea, diaphoresis and shock.

**Figure 8:**
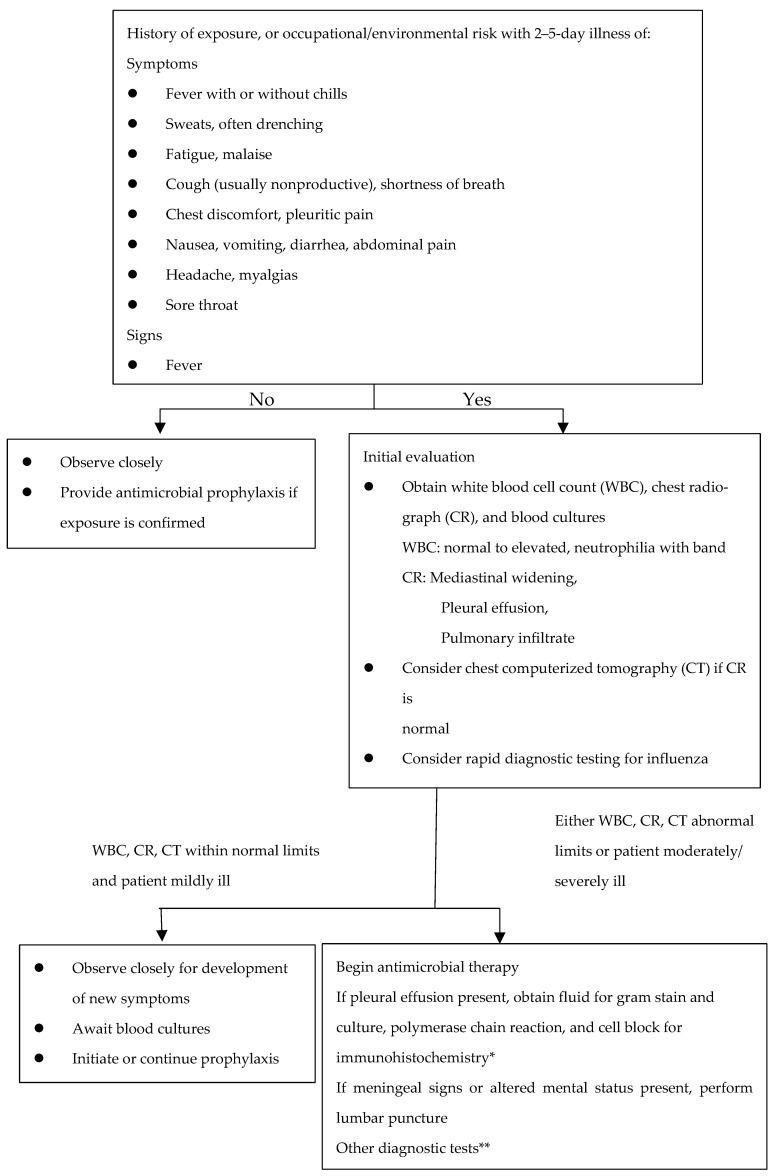
Recommended clinical evaluation for patients with possible inhalational anthrax. * Available through CDC or LRN. Cell block obtained by centrifugation of pleural fluid. ** Serologic testing available at CDC may be an additional diagnostic technique.

A triage algorithm published in the Morbidity and Mortality Weekly Report by the Centers for Disease Control and Prevention, which is in the public domain, has a slow timeline; thus, more frequent examinations need to be performed to help the wounded. This triage algorithm is shown in Figure 8. The first step is to check whether the high-risk wounded with exposure history have nonspecific symptoms listed in the first stage. If there are no symptoms, the wounded should continue to be observed; if exposure is determined to be asymptomatic, then antimicrobial prophylaxis should be performed.

In the case that first-stage symptoms are present, the wounded should be examined by conducting a leukocyte count, chest fluoroscopy, blood culture and CT. According to the examination results, the wounded should then be divided into two groups, those with WBC, CR, CT within normal limits and patient mildly ill and those with either WBC, CR, CT abnormal limits or patient moderately/severely ill; different treatment measures are undertaken for the two groups.

**Figure 9:**
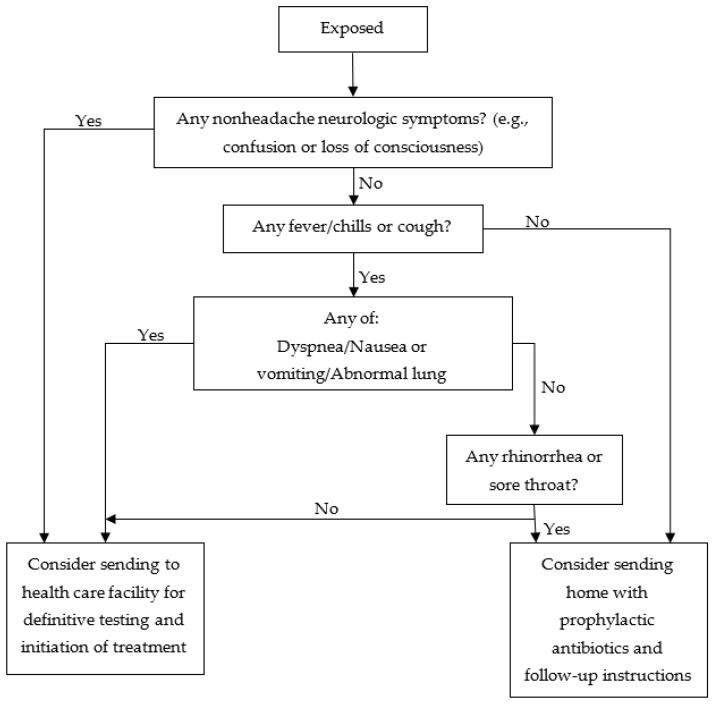
Three-tier screening protocol to identify potential early inhalational anthrax cases in the setting of a large-scale anthrax attack.

Sox, H.C. designed a triage algorithm for inhalational anthrax according to data collected on anthrax attacks in the United States in 2001; this triage algorithm is shown in Figure 9 [23]. The method starts by confirming the symptoms of the exposed wounded; this confirmation process is divided into three steps. According to whether the wounded have any non-headache neurological symptoms (fever, cough, dyspnea, nausea or vomiting, abnormal lung examination, rhinorrhea or sore throat), they are divided into two groups—namely, those who need to be sent to the hospital for further examination and those who can be given preventive antibiotics to take at home with follow-up guidance.

However, because there are few cases of anthrax bioterrorism, i.e., only 28 cases have been reported (1920–2003), this triage algorithm is the result of the study of only these 28 cases; thus, the efficiency of this set of triage algorithms is unknown [23].

Due to the rarity of inhaled anthrax cases, Hupert, N compared the clinical presentations of adult patients with anthrax in the English-language literature from 1880 through 2013 in 2019. A total of 408 case patients with inhalation, ingestion and cutaneous anthrax and primary anthrax meningitis and 657 control patients were included, and a checklist was developed for initial triage after an anthrax mass-exposure event. Seven indices of patients were mainly examined [29].

**Figure 10:**
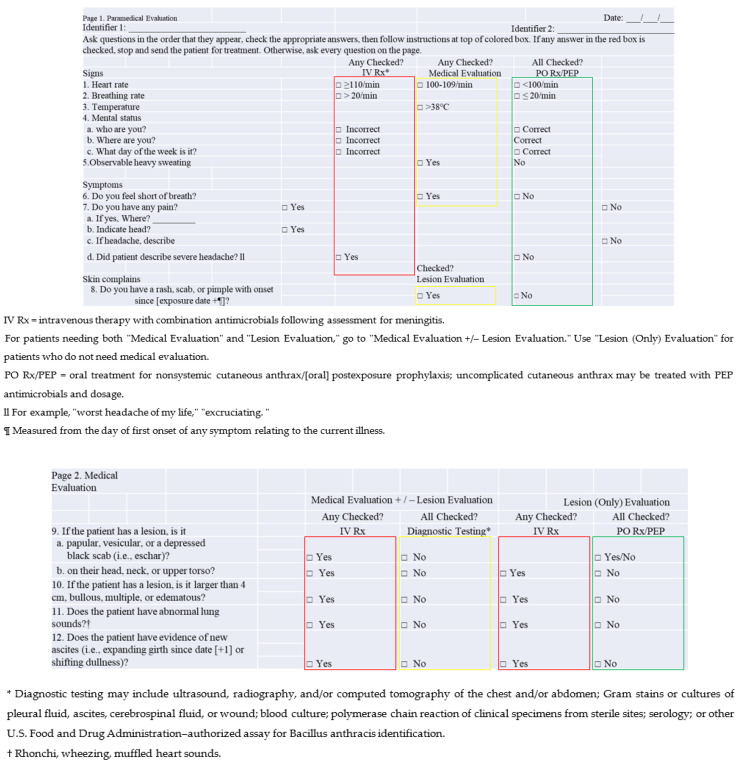
Checklist for initial triage after an anthrax mass-exposure event. Page 1: IV Rx (www.ncbi.nlm.nih.gov/pmc/articles/PMC5034857 accessed on 25 January 2022); PO Rx/PEP (www.ncbi.nlm.nih.gov/pmc/articles/PMC3901462 accessed on 25 January 2022); Page 2: U.S. Food and Drug Administration–authorized assay for Bacillus anthracis identification (https://wwwn.cdc.gov/nndss/conditions/anthrax/case-definition/2018 accessed on 25 January 2022).

The developed checklist is shown in Figure 10. The checklist is divided into two pages. The first page quantifies the physiological indices of the wounded and divides them into three categories according to the following values: intravenous therapy with combination antimicrobials following assessment for meningitis immediately; oral treatment for no systemic cutaneous anthrax/(oral) postexposure prophylaxis; and uncomplicated cutaneous anthrax treatment with PEP antimicrobials and dosage and medical evaluation.

The screening checklist combines easily collected vital sign data with brief questions on the patient’s mental function and symptoms; the only physical examination components (required for only 40% of the patients in our dataset) are the observation of cutaneous lesions, auscultation of the lungs and evaluation for ascites. The only instruments needed for screening are a timepiece, a thermometer and a stethoscope [29]. Therefore, the screening of the wounded can be completed faster.

### 3.5. Triage Algorithms for Mental or Psychosocial Problems Caused by Bioterrorism Events

In addition to producing toxic or infectious sequelae, a bioterrorist attack has the potential to cause widespread psychophysiological, social, behavioral and psychiatric morbidity [44]. Such widespread psychosocial casualties may lead to many people who fear that they have been attacked to visit the hospital for examination. In addition, panic can cause other diseases; if this happens, it may lead to a surge in demand for medical evaluation, which will disrupt the triage process and endanger the lives of those who have been truly attacked by bioterrorism [25].

If left undetected and untreated, fear-based signs and symptoms may be extremely debilitating and lead to chronic problems, with a risk of permanent damage to the brain’s locus coeruleus and stress-response circuits [24]. Thus, greater attention should be given to the triage of psychophysiological, social, behavioral and mental diseases caused by large-scale bioterrorism attacks.

Bracha, H.S. recommended that a fear and resilience (FR) checklist be included as an essential triage tool to identify those most at risk. There are four parts to the checklist. In Part 1, there is only one question that aims to determine whether the wounded has been exposed or infected. Part 2 contains seven questions that aim to assess whether the injured person has symptoms of infection. Part 3 has two questions that aim to evaluate the exposure status of the wounded’s family and whether the wounded is subject to medical evaluation. The checklist objectives of parts 1–3 are used to assist in the determination of whether a caller on a phone hotline or a citizen who has shown up at a triage-information center has likely been exposed/infected vs. likely not been exposed/infected.

**Figure 11:**
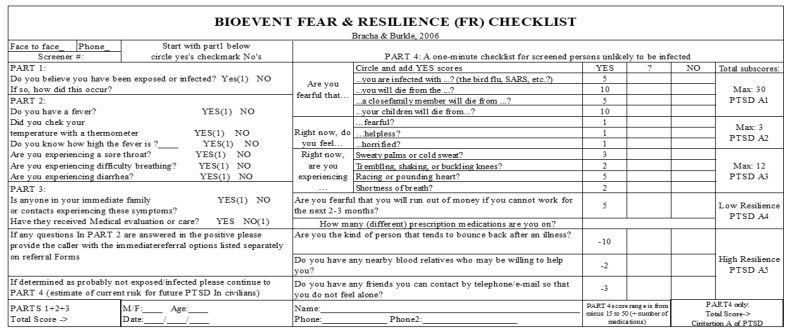
Bioevent fear and resilience checklist [24].

Part 4 consists of a one-minute checklist for screened persons who are unlikely to be infected; this part is drawn from Criterion A of PTSD determination. There are 17 questions in this part, including questions about emotional resilience, physical resilience and economic resilience. Part 4 scores range from a minimum of zero to a maximum of 50 (the higher the score is, the greater the risk is) [24]. The checklist is shown in Figure 11.

This checklist can not only be used to assess the severity of acute terror but can also identify most individuals who can be considered hyper-resilient and, therefore, could be asked to volunteer [24].

**Figure 12:**
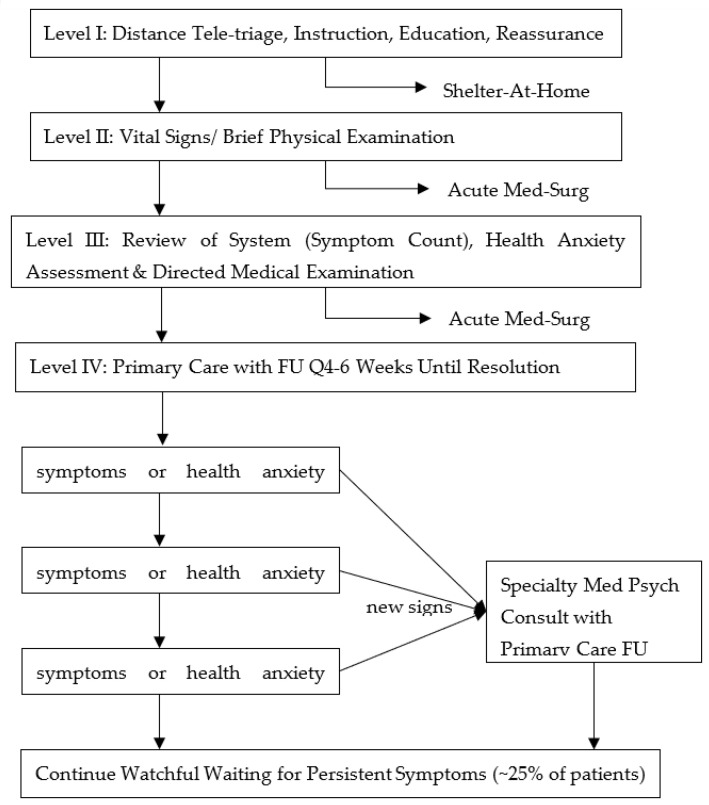
Triage algorithm for patients with possible idiopathic physical symptoms and acute health anxiety after a terrorist attack.

Engel, C.C. proposed a four-level triage algorithm for the health-care system’s response to surging demands for triage and management in the event of mass idiopathic illness and overwhelming health anxiety [25] as shown in Figure 12.

In this model, the assessment of the wounded is mainly performed by a clinician rather than by mental health-care professionals. First, tele-triage guidance should be undertaken. Second, vital signs and brief physical examinations should be performed for the urgently wounded. The third level aims to assess health anxiety according to standardized measurement tools. The location of the fourth level of care depends on the type of terrorist attack and treatment environment and the formulation of the local medical follow-up plan.

## 4. Discussion

Studies on triage algorithms for bioterrorism attacks are seriously insufficient. Most studies have proposed that attention be paid to triage algorithms but no specific triage algorithms are proposed. Some of the studies provided health-care providers regarding treatment but did not include information on diagnostic testing or triage decisions [45]. Only 10 studies proposed specific triage algorithms, and these can be divided into triage algorithms for most bioterrorism events, triage algorithms for anthrax attacks and triage algorithms for mental or psychosocial problems caused by bioterrorism events. We will explore each of them in more depth.

### 4.1. Triage Algorithms for Most Bioterrorism Events

When the specific type of bioterrorism attack is unknown, the general principle is to first judge whether the terrorist is a bioterrorism attack. Bioterrorism attacks are more covert, and the incidence may last up to several days after the attack. Once it is determined that a bioterrorism attack has occurred, it is necessary to determine the time and place of the attack as soon as possible, control the number of exposed and potentially exposed people, prevent infection and determine the type of biological agents used.

In the training course of dealing with mass casualties in Japan, the decontamination of the wounded was added to the triage process, and the criteria for selecting wet or dry decontamination are not sufficiently recognized [33]. Some studies proposed that all patients need the removal of clothing and a simple 5 to 6 min shower with soap and water. Only in rare circumstances may additional steps be necessary in decontamination, including gastric lavage, broncho-alveolar lavage, surgical removal of wound foreign bodies and the administration of activated charcoal, polyethylene glycol electrolyte solution and radioisotope-binding agents [34].

Some studies argue that the decontamination process should be placed before the triage process, i.e., that diagnosis, assessment, triage and emergency treatment should not occur until casualties arrive at the decontamination area [46]. Other studies argue that the decontamination process should be placed after the triage process, i.e., that all exposed and potentially exposed individuals should receive an initial brief triage performed by medical personnel in PPE before they are processed for decontamination [36]. Research on the effect of decontamination on bioterrorism attacks needs to continue. If decontamination is indeed effective in relation to bioterrorism attacks, then this process can be added to the triage algorithm. The choice of decontamination methods also requires consensus.

### 4.2. Triage Algorithm for Anthrax

The three triage algorithms for inhalational anthrax attacks are based on symptoms. The indicators that the three triage algorithms are concerned with at the beginning are shown in the Table 3 below.

Fever and dyspnea are the common screening symptoms used in the three methods; two methods suggested that excessive sweating and headache are also symptoms of an anthrax attack. If there are symptoms, then a further detailed examination is needed to determine whether the patient is infected with *Bacillus anthracis*. Some symptoms of inhaled anthrax are similar to those of ordinary influenza. The efficiency of distinguishing whether one is infected by anthrax according to one’s symptoms needs to be further verified [23].

Future research should focus on the efficiency of the triage of those with anthrax infection and those without anthrax infection according to symptoms. In addition, after a large-scale bioterrorism attack, many people who think they have been attacked may come to the hospital due to the impact of psychological factors. If they can pass a screening in advance and, thereby, allow the treatment of anthrax-based biological attacks to be given to the wounded who have indeed been attacked by anthrax, then the overall outcome may be better.

Children and pregnant women are populations that may require special consideration [47]. More attention could be paid to children and pregnant women who have been attacked by anthrax, and follow-up studies could be conducted on whether children and pregnant women have special symptoms when they are attacked by anthrax. In a recent systematic review of 20 natural cases, maternal and fetal mortality were shown to be high [48].

### 4.3. Triage Algorithms for Mental or Psychosocial Problems Caused by Bioterrorism Events

In addition to producing toxic or infectious sequelae, a bioterrorist attack has the potential to cause widespread psychophysiological, social, behavioral and psychiatric morbidity [44]. Even less is known about the psychological reactions to the threat of terrorist attacks that employ biological or chemical weapons (BCW) or the psychological impact of actual BCW terrorist events with large numbers of victims and an even greater number of covictims [49].

The psychological problems caused by bioterrorism attacks are different from those caused by ordinary car accidents. After bioterrorism attacks, a large number of people will feel panic and uneasiness, which makes people think that their workplace and even their community and home are no longer safe [49]. The mental health problems of medical personnel cannot be ignored. Once psychological problems of medical personnel occur, their presence will affect the order of treatment work and may cause adverse consequences.

Before the occurrence of large-scale bioterrorism attacks, certain measures, such as mental-health training, can be taken to reduce the possibility of psychological problems of personnel afterward [49]. The effectiveness of pretraining needs to be proven by follow-up research. After an incident, attention should be given to the psychological status of key personnel, such those with the loss of a loved one to the attack, proximity to the site, media exposure to the attack, a history of chronic psychiatric illness and a previous pattern of idiopathic physical symptoms. Such individuals include children, frail elderly patients, those lacking in social support, homeless people, first responders, health-care providers and patients who are disabled [25].

## 5. Limitations

First, there are few studies on large-scale bioterrorism attacks, and there are even fewer studies thar focus on triage in large-scale bioterrorism; therefore, the number of studies included in the current review was small. Second, due to the rarity of large-scale bioterrorism attacks, most of the existing studies cannot prove the efficiency of triage algorithms. They can only propose a general framework of a triage algorithm, and there is no conclusion about which algorithms are better than others.

## 6. Conclusions

This paper systematically summarized the existing triage algorithms that deal with large-scale bioterrorism attacks. The existing research can be divided into triage algorithms for most bioterrorism events, triage algorithms for anthrax attacks and triage algorithms for mental or psychosocial problems caused by bioterrorism events. There are four different triage algorithms for most bioterrorism events; however, there is no conclusion regarding which of the four methods is better. In the face of large-scale bioterrorism attacks, there are two key points for triage; the first is to identify the biological agents used in the attack, and the second is to improve the efficiency of injury detection and the classification of exposed people.

An anthrax attack is most likely to cause inhalational anthrax infection. Future research should improve the distinction between inhalational anthrax symptoms and common disease symptoms and improve the efficiency of triage measures. There are few studies on triage algorithms regarding the mental or psychosocial problems caused by bioterrorism events. Thus, more attention should be given to this issue. Medical staff should add psychological education to their usual training procedures.

## Figures and Tables

**Table 1:** Systematic search strategy.

	Search Terms		
	Medline	Web of Science	Scopus
**1. Set of entry criteria**		TS = (triage) OR TS = (triages)	
**2. Set of entry criteria**		TS = (“Bioterrorism”) OR TS = (Terrorism, Biological) OR TS = (Biological Terrorism)	
**3. Final search**	(((“Bioterrorism”[Mesh]) OR (Terrorism, Biological)) OR (Biological Terrorism)) AND ((“Triage”[Mesh]) OR (Triages))	1 AND 2	((TITLE-ABS-KEY(triage)) OR (TITLE-ABS-KEY(triages))) AND ((TITLE-ABS-KEY(Bioterrorism)) OR (TITLE-ABS-KEY(Terrorism, Biological)) OR (TITLE-ABS-KEY(Biological Terrorism)))

**Table 2:** Quality assessment (QA) of the included studies.

No.	Author Name	Year	Title	Total Score	Quality Assessment	Article Type	Triage Algorithm	Applicable Scenario
1	Burkle, F.M. [22]	2002	Mass casualty management of a large-scale……	4	L	Review	SEIRV Model	After a bioterrorism attack to disitiguish which patients requiring treatment.
2	Sox, H.C. [23]	2003	A Triage Algorithm for Inhalational Anthrax……	4	L	Editorial Material	Three-tier screening protocol to identify potential early inhalational anthrax cases in the setting of a large-scale anthrax attack	After a anthrax attack. Triage for people who have symptoms of inhalational anthrax.
3	Cone, D.C. [18]	2005	Mass casualty triage in the chemical, biological……	5	H	Review	Triage Algorithm Based on START and the GCS	In the event of a covert release of a biological weapon, overt release or when the source of a biological agent is ascertained
4	Bracha, H.S. [24]	2006	Utility of fear severity and individual resilience……	5	H	Original Research	BIOEVENT FEAR & RESILIENCE (FR)CHECKLIST	After a bioterrorism to identify individuals with excessive levels of fear.
5	Burkle, F.M. [24]	2006	Population-based triage management in response to……	4	L	Article	SEIRV Model	After a biological terrorism to disitiguish which patients requiring treatment.
6	Engel, C.C. [25]	2007	Terrorism, trauma, and mass casualty triage: How……	4	L	Review	Triage algorithm for patients with possible idiopathic physical symptoms and acute health anxiety after a terrorist attack	Healthcare systems’ response to surging demands for triage and management in the event of mass idiopathic illness and overwhelming health anxiety.
7	Melnick, A.L. [26]	2007	Biological, chemical, and radiological terrorism: ……	5	H	Book	Triage algorithms for inhalational anthrax and cutaneous anthrax	Clinical evaluation of persons with possible cutaneous anthrax and for patients with possible inhalational anthrax
8	Bond, W.F. [27]	2008	Testing the use of symptom-based terrorism triage……	6	H	Original Research	Attack algorithm and biologic agent algorithm	After a terrorism attack
9	Anan, H. [28]	2016	Development of Mass-casualty Life Support……	4	L	Special Report	Pre-decontamination Triage	Responding to alltypes of CBRNE disasters
10	Hupert, N. [29]	2019	Development and Performance of a Checklist……	7	H	Original Research	Checklist for Initial Triage After an Anthrax Mass Exposure Event	After a anthrax attack to identify anthrax case patients needing intravenous antibiotics.

L: Low and H: High.

**Table 3:** Comparison of indicators concerned by the three triage algorithms.

Triage Algorithms	A Triage Algorithm for Inhalational Anthrax	Checklist	Triage Algorithm from Centers for Disease Control and Prevention, Public Domain, of Morbidity and Mortality Weekly Report
**Symptoms**	non-headache neurologic symptoms	heart rate	fever with or without chills
	fever, chills or cough	respiratory rate	sweats, often drenching
	dyspnea, nausea or vomiting, abnormal lung examination	temperature	fatigue, malaise
		mental status	cough shortness of breath
		diaphoresis	chest discomfort, pleuritic pain
		dyspnea	headache, myalgias
		severe headache	sore throat
		characteristic skin lesions	fever
		abnormal lung sounds	

## Data Availability

Data sharing not applicable. No new data were created or analyzed in this study. Data sharing is not applicable to this article.

## Supplementary Materials

The following supporting information can be downloaded at: https://www.mdpi.com/article/10.3390/ijerph20065070/s1, PRISMA 2020 checklist.
